# Development of Construction Material Using Wastewater: An Application of Circular Economy for Mass Production of Bricks

**DOI:** 10.3390/ma15062256

**Published:** 2022-03-18

**Authors:** Sajid Ghafoor, Abdul Hameed, Syyed Adnan Raheel Shah, Marc Azab, Hamza Faheem, Muhammad Faisal Nawaz, Fahad Iqbal

**Affiliations:** 1 Department of Civil Engineering, Pakistan Institute of Engineering and Technology, Multan 66000, Pakistan; 2 College of Engineering and Technology, American University of the Middle East, Kuwait; 3 Department of Mechanical and Structural Engineering and Materials Science, University of Stavanger, NO-4036 Stavanger, Norway

**Keywords:** wastewater, construction, brick, eco-friendly, resource management

## Abstract

Water is one of the necessary ingredients for construction materials. Billions of gallons of clean water are wasted during the development of fired clay bricks. Similarly, the waste of clean water is a global issue. In this study, we develop fired clay bricks with the help of wastewater for the first time and compare these with clay bricks produced using groundwater, which is the conventional method. Both destructive (i.e., compressive strength (CS)) and non-destructive (i.e., ultrasonic pulse velocity (UPV)) tests are conducted on all fired clay brick specimens as per the American Society for Testing and Materials (ASTM). Physical (i.e., dimensions) and durability (water absorption, efflorescence, etc.) tests are also conducted. All kinds of brick satisfied the standard requirements of physical and durability characteristics. Similar or better strength of bricks were achieved using wastewater. The study concludes that the testing results of wastewater bricks were significantly 15–25% higher compared with groundwater-fired clay bricks. A large amount of wastewater can be used to develop bricks, and clean water can be saved to attain circular economy goals. Therefore, this study will help not only in developing low-cost bricks but also in saving clean water.

## 1. Introduction

A combination of water and clayey soil is essential to produce fired clay bricks. First, the bricks are dried through sun heat then put into a kiln for firing purposes [1]. Clay bricks have been produced at waterfronts where suitable soil could be found, and they are the oldest and most used building material [2,3]. They are an ancient material that is easily obtainable for construction in the market is the soil because they are ecological, reasonable, and frequently existing [4]. 

On the other hand, humans prefer materials that are cost-effective and usually available for shelter erection. Hence, people from various regions and time eras preferred timber, stone, and mudbrick as elementary edifice materials. Approximately 1.3 trillion bricks are manufactured each year worldwide [5]. Fired clay and mud bricks remain in their ordinary forms in contrast with timber and stone because they require a technical and preparatory stage. Hence, bricks are an initial edifice material that are produced according to specific size requirements. Moreover, suitable structural methods and forms were settled according to the ordinary properties of these constituents [6,7]. Due to the less weight, easier preparation clay bricks were frequently utilized in ceilings as compared to stone because the orientation of rows and thickness control is easier [8]. 

From ancient times, the production of bricks has been performed in the Egyptian, Mesopotamian, and Roman eras [3,7]. In Mesopotamia, a great deal of applications were found in Syria and Iraq earlier fourth century [9,10,11]. Throughout the Roman periods, the consumption of clay bricks was increased due to its better structural properties [3]. In the fourth century, BC Lydians in Anatolia manufactured the first foremost fired bricks [12]. 

A well-known example of the combination of brick and stone was later detected in Seljuk architecture. Moreover, Byzantines and tiles in Anatolia added enhancements in the use of bricks [7]. Initial standards of production were announced during the Ottomans, Until the industrial revolution, no more advancement was seen in brick production [12]. Until the mid of 20th century, in Anatolia, the brick-making technology of the Ottomans remained in use. Hence, accomplished data relevant to brick manufacturing is possible [13]. 

An extensive river basin was occupied the ancient civilizations that were also used for the creation and manufacturing of alluvial sediments and bricks. Clay bricks are widely used as a building material due to their better durability, compressive strength, fire and weathering resistance, and good thermal and soundness characteristics. The ability to build a high-rise structure is also due to brick manufacturing. As well as sun-dried brick gains less importance compared to fired bricks [14]. Researchers have evaluated the architectural applications of clay brick and studied their chemical and mechanical characteristics [3,4,8,14,15,16,17,18,19]. However, few studies elaborate on the production methods of brick in detail. 

**Figure 1:**
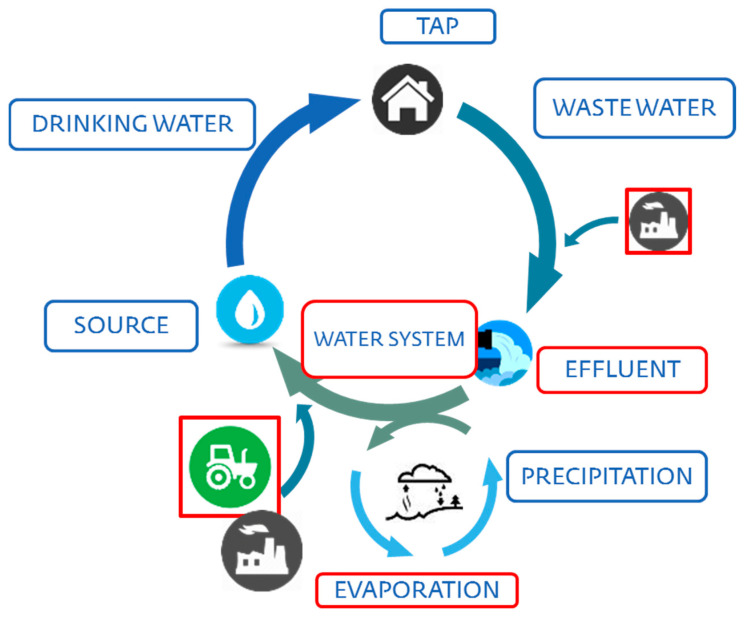
Water management cycle [20].

The water resource management cycle is about dealing with all types of water resources and using wastewater for a suitable assignment is related to the circular economy. The cycle related the utilization of water at the domestic level to industries as shown in Figure 1 [20].

Usually, in the construction process, potable water is used, which can be replaced with wastewater. This process will not only save clean water but will also help to clean wastewater by evaporation of wastewater during the brick burning process.

The primary goal of this article is to overcome this deficiency in the literature. Previously wastewater has only been used in the development of concretes [21,22,23,24,25,26,27,28,29,30,31], and only olive oil mill wastewater has been tested for the development of clay brick [32,33]. However, mass production is not possible with such sources of water in the case of bricks. Thus, domestic wastewater, which is the largest source of wastewater around the world is proposed to be utilized for the mass production of clay bricks. This process will work in parallel to the circular economy concept.

## 2. Research Methodology

**Figure 2:**
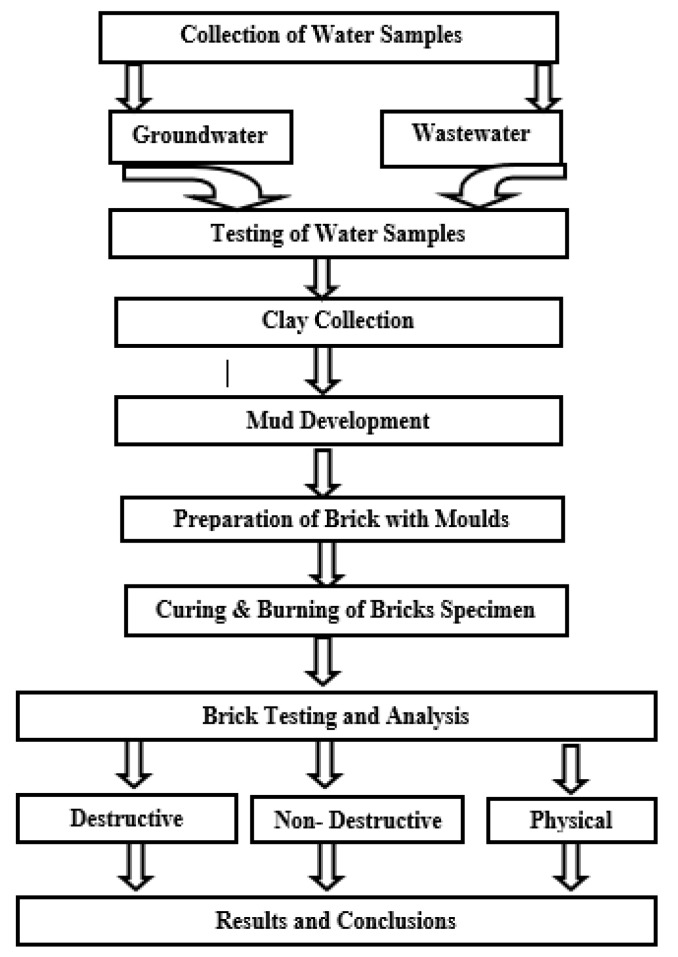
Methodological framework.

The itemized strategic system for the development and strength investigation of created material [21,22,24,34] using different water resources is given in Figure 2.

As per the established concept of concrete development and strength analysis, the following steps were performed to develop the concrete with help of groundwater, surface water, and wastewater, and later, they were tested according to standard procedures [21,22,24,34].

### 2.1. Manufacturing of Bricks

The method involved with assembling brick blocks is performed in a few phases. These are recorded beneath. Each stage has its particular significance.

P 1. Choice of the water samples for brick development.

P 2. Choice of the reasonable kind of brick earth.

P 3. Arrangement and tempering of mud.

P 4. Forming or embellishment of brick block units.

P 5. Drying of formed brick blocks.

P 6. Cooling and burning of brick blocks.

Good quality building bricks cannot be made from every type of earth or soil. As a general statement, it may be said that soil (earth), which contains four parts of clay, and one part of sand, is suitable for making bricks.

### 2.2. Basic Materials

#### 2.2.1. Clay

**Figure 3:**
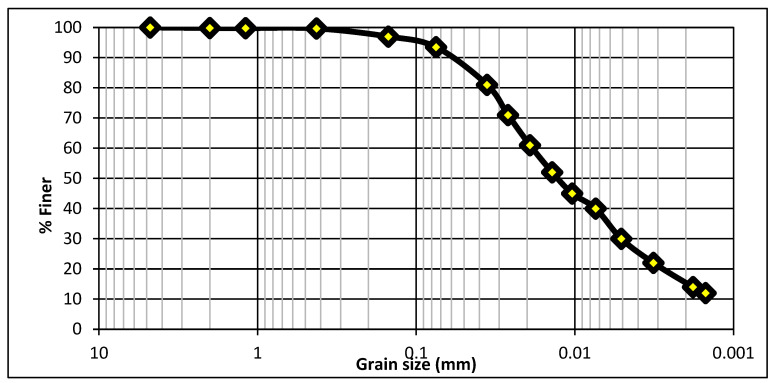
Particle size distribution curve.

The primary fine-grained natural material that is used for brick specimen preparation is clay (soil), which is locally and easily available. Clay also contains traces of metal oxides and organic matter. The nature of clay is plastic because it shows non-plastic, brittle, and hard properties upon drying or firing due to the water content. The clay used in this study was taken from the kiln situated in Multan City, Pakistan. Due to the mechanical and durability characteristics of fired clay bricks, the sieve analysis of soil is very significant, and the porosity of brick specimens is affected due to the size of soil particles [35,36]. The particle size distribution curve [37] is shown in Figure 3.

The color of clay can depend upon the content of raw materials found in soil with the help of sieve analysis. Many occurring rocks and deposits in the world also contain silt and clay. Dissimilarity among clay and silt varies by discipline because silt contains large particle diameters compared with clay. The geological composition of raw materials in the soil is listed in Table 1.

#### 2.2.2. Mixing Water

Water is an important constituent for brick manufacturing. In this experimental work, two types of water were used; the first is groundwater or tap water that was obtained from 250 feet under the ground level; the second is wastewater that was obtained from local source. On daily basis, this sewage water is disposed of into the Naubahar Canal as wastewater. At present, the wastewater is collected from this disposal point. The chemical analysis tests of water, such as the total suspended solids (T.S.S), pH, conductivity, total dissolved solids (T.D.S), etc. are to be conducted on all types of water samples to investigate their characteristics. The results of this analysis according to World Health Organization (WHO) [38] and Environmental Protection Agency (EPA) [39] are shown in Table 2.

The results of the chemical analysis indicated that the wastewater values were beyond the safe limits described by the WHO and EPA for drinking water. On the other hand, treatment and disposal of this harmful water is a primary issue that is faced by the whole world. Many techniques have been adopted to overcome and mitigate the effects of these waters on the environment. Many environmental and economic benefits are easily conquered after the utilization of this harmful water into bricks production to save a million liters of groundwater or drinking water that is used for daily brick production.

### 2.3. Mix Design and Sample Preparation

The mixed design criteria for brick preparation involve a combination of clay and water. Approximately 25% of water by total weight of clay is taken for the mixing of clay for brick preparation. First, the lumps of clay are broken to ensure that no lumps are found in the clay. After this, the water is added into dry clay and left in the mixture for 2–3 h to fill the voids for homogenous mixing. The brick mold size was 228 mm × 114 mm × 76 mm in this study [40]. 

**Figure 4:**
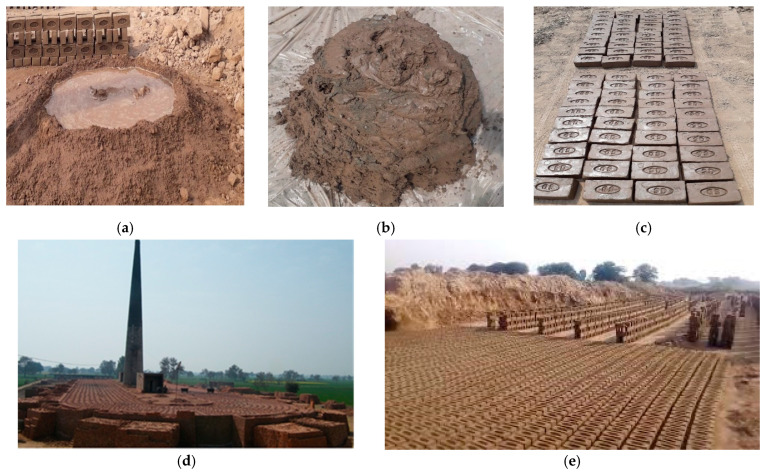
Burnt fired clay manufacturing: (**a**) Wet mixing and voids filling of clay. (**b**) Clay lump. (**c**) Sun drying of demolding specimens. (**d**) Fired in standard Hoffmann kiln. (**e**) Mass production of clay bricks.

After homogenous mixing, the clay lump is put into the mold, and sand is used for lump coating to avoid sticking to the mold. For drying purposes, the bricks are dried for 2–3 days by the heat of the sun then put into a kiln for 10 days to burn at a temperature of more than 1000 °C. For firing and production purposes, the common Hoffmann kiln is used in the manufacturing process as shown in Figure 4. 

The water–clay ratio is kept constant at 0.25 for all types of brick preparation. Thus, a total of 56 samples (20 (destructive + non-destructive testing—Bricks) + 36 (Durability and Physical Analysis—Bricks)) were developed. 

### 2.4. Testing

After burning, the following tests were conducted, on the burnt fired clay specimens.

#### 2.4.1. Destructive Testing

**Figure 5:**
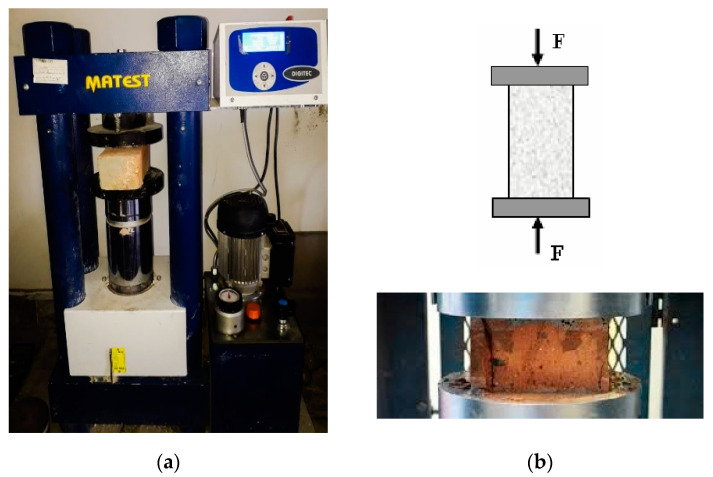
(**a**) Compression machine with brick. (**b**) Compressive strength concept.

In destructive testing, the compressive strength test was conducted on brick specimens as per ASTM [41]. The load was applied in the direction of depth of burnt clay bricks, and the loading rate was 2.5 kN/s. Before testing, the specimens were capped on both sides with the help of gypsum and left for 24 h for drying purposes. The compression machine is shown in Figure 5.

#### 2.4.2. Non-Destructive Testing

**Figure 6:**
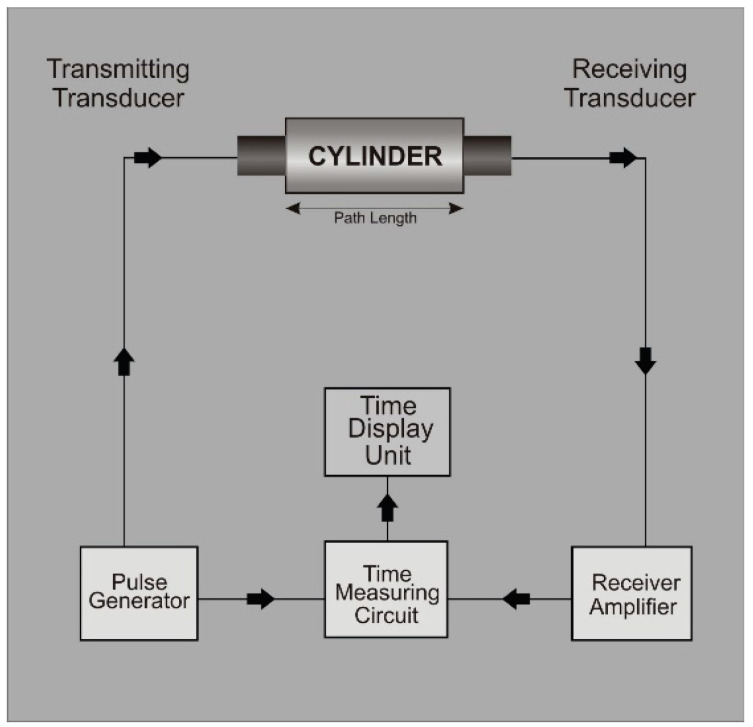
Ultrasonic pulse velocity working flow diagram [43].

Another type of testing used in this study is non-destructive testing. In this testing, an ultrasonic sonic pulse velocity test was applied along the length of specimens. Essentially, with the help of this test, the flaws can easily be measured in the brick specimen [40]. Two transducers were used: one is the transmitting transducer, and the other is the receiving transducer. The 50 kHz transducers were used on opposite sides of the specimen to measure the pulse velocity of these specimens [42]. A coupling agent was used for better results. The basic working flow diagram is shown in Figure 6.

The test was conducted according to ASTM [44], and the velocity was found with the help of the equation given below:

(1) $$V=\frac{L}{T}$$

where V = Pulse velocity (m/s); L = Path length (m); and T = Time (µs).

#### 2.4.3. Durability Testing

The durability or physical characteristics of bricks are more important than the mechanical properties. The behavior of specimens against these tests shows the quality and strength properties of these specimens. The following tests are performed on brick specimens according to ASTM and BIA [41,45] and are given below:

- For dimension purposes, the brick is laid along the length, width, and height to determine the standard dimensions. In this test, twenty bricks are taken randomly, and then with the help of measuring tape, the dimensions are measured.
- For the soundness test, randomly, two bricks are taken from the batch, and then they are struck relative to each other. For a good quality brick, a bell, or ringing sound, is produced when they have struck each other.
- For the hardness test, any brick is taken from the batch, and with the help of a fingernail, a scratch is made on the brick surface. If no scratch impression is visible on a brick surface, then the brick is hard and durable.
- For the impact test, the brick falls from approximately 1 m in height, and after falling, the quality of brick is examined regarding whether it is broken or not.
- For the water absorption test, before the test, the specimen is completely dried and then immersed in tap water for 24 h. After 24 h, the bricks are removed, and the weight of these bricks is individually measured. The main factor that affects the durability properties of the bricks is water absorption [46].
- For the efflorescence test, the specimen is partially immersed in water to determine the existence of soluble salts in brick and then rests in the water at a depth of 25 mm for 24 h. After 24 h, the brick is removed from the water and then observed [41].

## 3. Results and Discussions

### 3.1. Analysis of Physical and Durability Properties

All the physical and durability properties, such as the impact, dimensions, soundness, hardness, water absorption, and efflorescence of specimens are investigated according to ASTM. The dimensions of every type of brick specimen are verified, and the impact analysis is done by falling the brick from a 1 m height. All the bricks are verified against hardness and soundness. The physical analysis is shown in Table 3.

**Figure 7:**
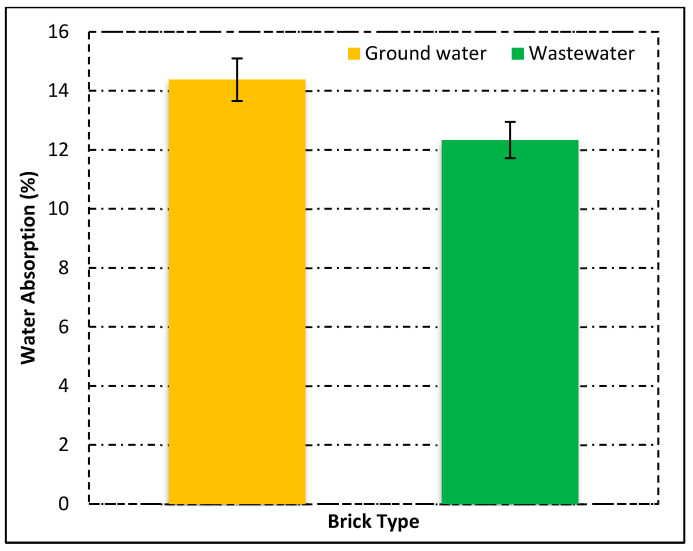
Water absorption of fired clay bricks.

After the physical analysis, the main property of any type of brick is its water absorption because the durability is primarily dependent upon the water absorption [46,47,48]. In this experimental study, the water absorption of all bricks was measured according to ASTM [41]. Figure 7 shows the absorption analysis of all types of bricks.

All the bricks absorbed less than 17% of water as shown in Figure 7. ASTM [49] stated that the water absorption for severe and moderate weather resistance must not be higher than 17% and 22%, respectively, for fired clay brick masonry [49]. On the other hand, a 20–30% maximum water absorption was described by a previous researcher for brick specimens [46,50,51]. The other property that affects durability is the appearance of soluble salts on the surface of bricks. This is also considered as an aesthetic defect [52].

After 24 hr. of observation, moderate soluble salts appear on the brick specimens, and the chief element that causes efflorescence is Cao (Calcium oxide) [53]. Almost 9% of Cao is present in the clay.

### 3.2. Analysis of Destructive Results

**Figure 8:**
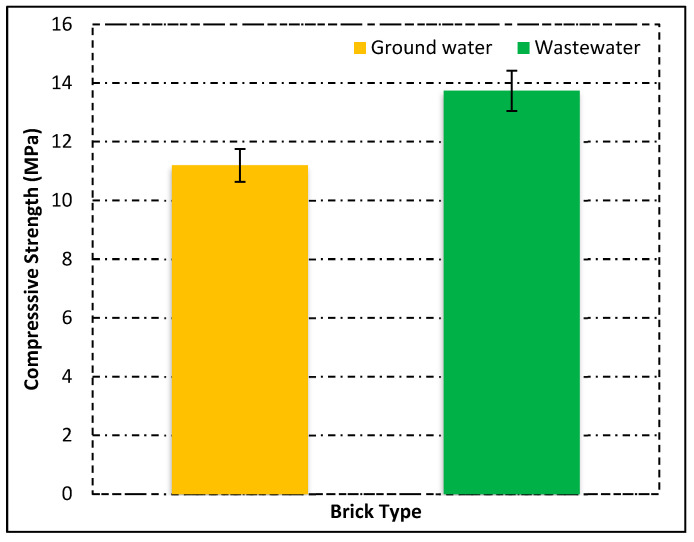
Compressive strength of fired clay bricks.

In this analysis, the compressive strength was observed for two different types of bricks. Compressive strength is the major property of the brick specimen that tells us about the strength behavior of burnt-fired clay bricks. The analysis is shown in Figure 8. 

The figure clearly shows that the wastewater bricks had high strength as compared with groundwater types of brick. The strength of wastewater bricks was 19% more than groundwater brick. The compressive strength of groundwater bricks was approximately 11.50 MPa, and thus all these bricks lie in the first-class brick category. The strength analysis results of wastewater bricks were better than groundwater bricks.

### 3.3. Analysis of Non-Destructive Results

**Figure 9:**
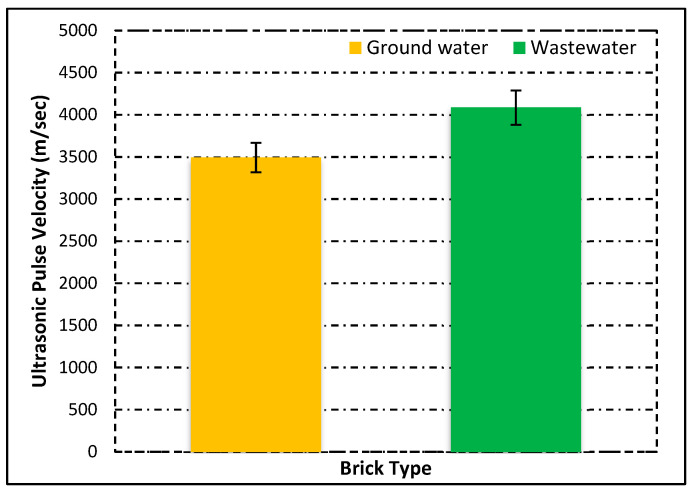
Ultrasonic Pulse Velocity of fired clay bricks.

In this analysis, the results of the ultrasonic pulse velocity test are analyzed, and the brick quality is to be examined. The test is applied on all types of bricks. Before the test, the bricks are completely dry, and then a coupling agent is used for better and more accurate results. Figure 9 shows the UPV results. 

This figure shows that the groundwater has a low UPV value as compared with wastewater bricks. However, this test is also very helpful to determine the quality of the structure element because the destructive analysis is very costly and time-consuming [54]. However, the non-destructive analyses, such as the ultrasonic pulse velocity, are easy to operate and economical to determine the voids and strength of concrete and brick also [55]. Previous work has shown that, if the UPV is higher than 3500 m/s, the brick specimen is durable and of good quality.

On the other hand, if the UPV is lower than 1000 m/s, then the brick is not recommended for use as a structural element because they are not durable, and many voids are available [56]. However, if the value lies in the range of 1000–3500 m/s, then both compressive and UPV tests are applied on a specimen to determine their performance against durability [57]. However, in our experimental study, all the values of UPV were equal and greater than 3500 m/s, and the values were between 3500 and 4100 m/s. This indicates that all types of bricks are durable and strong for use in brick masonry. No negative influence of wastewater was found for these bricks.

### 3.4. Statistical Analysis

**Figure 10:**
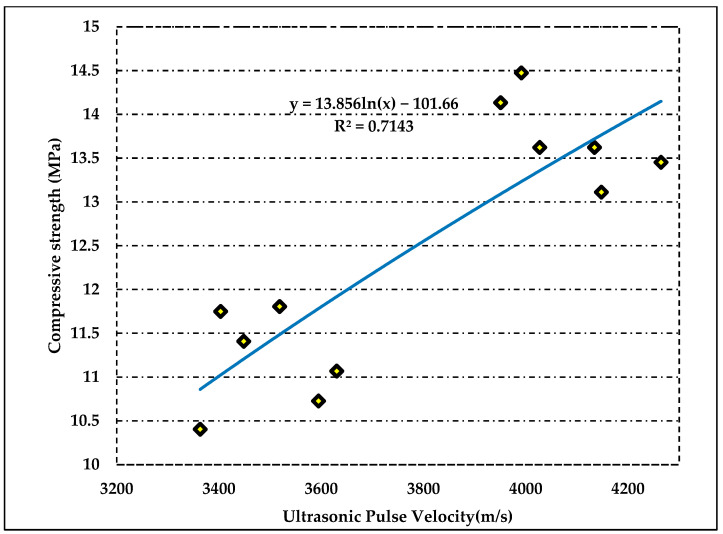
Regression analysis between destructive and non-destructive testing.

After destructive and non-destructive testing and water quality analysis, a detailed statistical analysis of results was conducted. The relationship was developed between destructive and non-destructive testing with the help of regression analysis. Figure 10 shows the regression analysis between destructive and non-destructive tests.

The value of R^2^ is 0.71, which shows a strong relationship among these tests. This relation is helpful for estimating the compressive strength of fired clay bricks. Overall, the behavior of compressive strength directly proportional to the ultrasonic pulse velocity. This can be proven through this linear regression line and model equation. Thus, the relationship successfully developed among destructive and non-destructive testing.

### 3.5. Wastewater Requirment Analysis

In Asian regions, different sizes of bricks are used and, at the local level, bricks of the dimensions 228 mm × 114 mm × 76 mm (length × depth × height). During the development of a brick with the above mentioned size, 0.7 L of water is utilized. During the process of ground molding—usually at a brick kiln setup—750 bricks/day are prepared. Thus, according to a simple calculation, 750 × 0.7 = 525 L of water are utilized. 

Therefore, during the process of brick manufacturing that quantity of clean water can be saved and also be replaced with wastewater. Previous research has discussed the utilization of wastewater for concrete development; however, this study is the first on wastewater utilization for mud brick development. This research has a strong footing as billions of bricks are produced worldwide, and similarly billions of liters of clean water can be saved if wastewater can be utilized.

## 4. Summary and Conclusions

In this experimental study, a sustainable and eco-friendly brick material was developed by adding various types of waters i.e., groundwater and wastewater. After chemical analysis, many parameters showed critical values that were very harmful for the environment and humans. Commonly used clay bricks are efficiently made with various types of water resources. For experimental investigation, both the destructive and non-destructive tests were conducted and analyzed. The results demonstrated extraordinary consequences when replacing groundwater with wastewater. The results obtained from wastewater bricks were approximately 19% better than ground surface water bricks. 

This verifies the positive impact of wastewater on the strength development of fired clay bricks. The UPV results also prove that the mixing and preparation of fired clay bricks with wastewater is helpful to produce structural and durable construction materials. On the other hand, the physical and durability analyses also gave acceptable values, which also proves the effective production of wastewater bricks. The water absorption of wastewater bricks was only 12%, which strongly satisfies the criteria of ASTM C-62. In the end, the relationship was investigated among destructive and non-destructive testing outcomes with the help of regression analysis. 

The equation developed through this model had a strong R^2^ of 0.71. After experimental and theoretical analysis, we concluded that wastewater can successfully utilized for the mass production of bricks to achieve circular economy goals. Untreated wastewater can cause diseases and make the whole environment dirty and polluted. 

This is a ecological idea to use this dirty water in applications of civil engineering. This technique is also helpful for sustainable development, which is a primary agenda of the entire world. Through the production of bricks with wastewater, a million gallons of sub-surface water savings can occur. Due to the odor and other problems, these bricks can be used for boundary walls, flooring, and another non-structural elements.

## 5. Future Recommendations

With the successful manufacturing of fired clay bricks through the help of ground water and wastewater, the experimental outcomes revealed that wastewater can work as a replacement for groundwater. For further studies, scholars can utilize a different source of wastewater (i.e., sewage plants/wastewater treatment plants) for the development of fired black bricks and conduct research on the impact analysis of wastewater chemical composition on the strength of concrete. 

Similarly, researchers can conduct a study that utilizes this wastewater for the development of distinct kinds of bricks (e.g., fly ash brick, sand-lime bricks, concrete blocks and bricks, etc.). Furthermore, there may be some adverse health outcomes and health risks associated with the use of such bricks made with wastewater; however, after burning at more than 1000 °C, the majority of harmful factors are expected to be nullified.

## Figures and Tables

**Table 1:** Geological composition of soil.

Type of Particle	Percentage (%)
Gravel	0
Sand	6.51
Silt	79.49
Clay	14

**Table 2:** Chemical analysis results of water samples.

Parameters	Units	Maximum Allowable Limit	Ground Water	Wastewater
pH	N/A	6.8–8.5 WHO	7.8	7
T.D. S	mg/L	1000 WHO	407	880
T.S. S	mg/L	150 EPA	33	189
Turbidity	NTU	10 WHO	1.93	88
Bicarbonates	mg/L	1000 WHO	160	450
Conductivity	micro-S/cm	1000	712	1418
Hardness	mg/L	100 WHO	300	410
D.O	mg/L	4–7 EPA	5.7	4.3
C.O. D	mg/L	150 EPA	24	430
B.O. D	mg/L	80 EPA	17	301
Alkalinity	m.mole/L	EPA	3.2	9
Fluoride	mg/L	20 (EPA)	0.15	0.22

Note: Limit for drinking water.

**Table 3:** Physical analysis of Brick Specimens—References for Parameter Selection only [45].

Physical Parameter	Brick Type	
	Ground Water	Wastewater
Impact	✓	✓
Soundness	✓	✓
Hardness	✓	✓
Dimensions	✓	✓

✓ = According to relative Standard.

## Data Availability

Data will be available on suitable demand.

## References

[1] Dalkılıç N., Nabikoğlu A. (2017). Traditional manufacturing of clay brick used in the historical buildings of Diyarbakir (Turkey). Front. Arch. Res..

[2] Ward-Perkins J.B. (1994). Roman Imperial Architecture.

[3] Fernandes F.M., Lourenço P.B., Castro F. (2010). Ancient Clay Bricks: Manufacture and Properties. Materials, Technologies and Practice in Historic Heritage Structures.

[4] Ren K., Kagi D. (1995). Upgrading the durability of mud bricks by impregnation. Build. Environ..

[5] Baseer S.K. (2016). Building Industry. https://dailytimes.com.pk/92270/brick-industry/.

[6] Oppenheim A.L. (2013). Ancient Mesopotamia: Portrait of a Dead Civilization.

[7] Bakırer (1981). Pre-Seljuk and Seljuk Period Anatolia—The Use of Brick in Architecture.

[8] Yavuz A.Ö., Sağıroğlu Ö. (2016). Reviewing the Bricks Used in The Traditional Architecture with The Shape Grammar Method. Gazi Univ. J. Sci..

[9] Moorey P.R.S. (1999). Ancient Mesopotamian Materials and Industries: The Archaeological Evidence.

[10] Saner T. (2005). Materials and Architecture in the Greek and Roman Periods. Material and Architecture in Anatolia from the Past to the Future.

[11] Ahunbay M. Early Period of Diyarbakır-Amida Walls. Proceedings of the International Diyarbakir Walls Symposium.

[12] Görçiz G. (1996). Brief History of Brick and Tile Industry1, Brick and Tile Industry.

[13] Binan C. (2005). Comments on Architecture, Material and Building Production in the Context of Transportation and Accommodation Structures in Anatolia. Proceedings of the Symposium/UIA 2005 XXIInd World Architecture Congress.

[14] Shakir A.A., Mohammed A.A. (2013). Manufacturing of Bricks in the Past, in the Present and in the Future: A state of the Art Review. Int. J. Adv. Appl. Sci..

[15] Dondi M., Marsigli M., Venturi I. (1999). Microstructure and mechanical properties of clay bricks: Comparison between fast firing and traditional firing. Br. Ceram. Trans..

[16] Lopez-Arce P., Garcia-Guinea J. (2005). Weathering traces in ancient bricks from historic buildings. Build. Environ..

[17] El-Gohary M., Al-Naddaf M. (2009). Characterization of bricks used in the external casing of roman bath walls “Gadara-Jordan”. Mediterr. Archaeol. Archaeom..

[18] Lourenço P.B., Fernandes F., Castro F. (2010). Handmade Clay Bricks: Chemical, Physical and Mechanical Properties. Int. J. Arch. Heritage.

[19] Sağın E.U. (2017). Properties of Roman period building bricks in Anatolia. J. Fac. Eng. Archit. Gazi Univ..

[20] KWR (2017). Closing the Water Cycle by Reusing Treated Wastewater. https://www.kwrwater.nl/en/actueel/closing-the-water-cycle-by-reusing-treated-wastewater/.

[21] Ramkar A., Ansari U. (2016). Effect of treated waste water on strength of concrete. J. Mech. Civ. Eng..

[22] Shahidan S., Senin M.S., Kadir A.A., Yee L.H., Ali N. (2016). Properties of Concrete Mixes with Carwash Wastewater. Proceedings of the MATEC Web of Conferences.

[23] Gadzama E., Ekele O.J., Anametemfiok V.E., Abubakar A. (2015). UEffects of sugar factory wastewater as mixing water on the properties of normal strength concrete. Int. J. Sci. Environ. Technol..

[24] Wegian F.M. (2010). Effect of seawater for mixing and curing on structural concrete. IES J. Part A: Civ. Struct. Eng..

[25] Alaejos P., Bermúdez M.A. (2011). Influence of Seawater Curing in Standard and High-Strength Submerged Concrete. J. Mater. Civ. Eng..

[26] Silva M., Naik T.R. Sustainable use of resources–recycling of sewage treatment plant water in concrete. Proceedings of the Second International Conference on Sustainable Construction Materials and Technologies.

[27] Asadollahfardi G., Mahdavi A.R. (2019). The feasibility of using treated industrial wastewater to produce concrete. Struct. Concr..

[28] Varshney H., Khan R.A., Khan I.K. (2021). Sustainable use of different wastewater in concrete construction: A review. J. Build. Eng..

[29] Taherlou A., Asadollahfardi G., Salehi A.M., Katebi A. (2021). Sustainable use of municipal solid waste incinerator bottom ash and the treated industrial wastewater in self-compacting concrete. Constr. Build. Mater..

[30] Farid H., Mansoor M.S., Shah S.A.R., Khan N.M., Shabbir R.M.F., Adnan M., Arshad H., Haq I.-U., Waseem M. (2019). Impact Analysis of Water Quality on the Development of Construction Materials. Processes.

[31] Raza A., Shah S.A.R., Kazmi S.N.H., Ali R.Q., Akhtar H., Fakhar S., Khan F.N., Mahmood A. (2020). Performance evaluation of concrete developed using various types of wastewater: A step towards sustainability. Constr. Build. Mater..

[32] Mekki H., Anderson M., Amar E., Skerratt G.R., BenZina M. (2006). Olive oil mill waste water as a replacement for fresh water in the manufacture of fired clay bricks. J. Chem. Technol. Biotechnol..

[33] Mekki H., Anderson M., Benzina M., Ammar E. (2008). Valorization of olive mill wastewater by its incorporation in building bricks. J. Hazard. Mater..

[34] Meena K., Luhar S. (2019). Effect of wastewater on properties of concrete. J. Build. Eng..

[35] Elert K., Cultrone G., Navarro C.R., Pardo E.S. (2003). Durability of bricks used in the conservation of historic buildings—influence of composition and microstructure. J. Cult. Heritage.

[36] UNCHS (1988). Compendium of Information on Selected Low-Cost Building Materials, UN Centre for Human Settlements (Habitat), Nairobi. https://digitallibrary.un.org/record/73926?ln=fr.

[37] (2007). Standard Test Method for Particle-Size Analysis of Soils.

[38] WHO (2011). Guidelines for Drinking Water Quality Criteria.

[39] EPA (2018). National Standards for Drinking Water Quality, in Environmental Protection Agency.

[40] Kazmi S.M., Abbas S., Saleem M.A., Munir M.J., Khitab A. (2016). Manufacturing of sustainable clay bricks: Utilization of waste sugarcane bagasse and rice husk ashes. Constr. Build. Mater..

[41] (2003). Standard Test Methods for Sampling and Testing Brick and Structural Clay Tile.

[42] Bogas J.A., Gomes M.D.G., Gomes A. (2013). Compressive strength evaluation of structural lightweight concrete by non-destructive ultrasonic pulse velocity method. Ultrasonics.

[43] Qurashi M.A., Shah S.A.R., Farhan M., Taufiq M., Khalid W., Arshad H., Tayyab M., Shahzadi G., Waseem M. (2019). Sustainable Design and Engineering: A Relationship Analysis between Digital Destructive and Non-Destructive Testing Process for Lightweight Concrete. Processes.

[44] (2009). Standard Test Method for Pulse Velocity through Concrete.

[45] BIA (2004). Manufacturing, Classification, and Selection of Brick.

[46] Phonphuak N. (2013). Effects of additive on the physical and thermal conductivity of fired clay brick. J. Chem. Sci. Technol..

[47] Zhang L. (2013). Production of bricks from waste materials–A review. Constr. Build. Mater..

[48] Velasco P.M., Ortíz M.M., Giró M.M., Velasco L.M. (2014). Fired clay bricks manufactured by adding wastes as sustainable construction material—A review. Constr. Build. Mater..

[49] (2017). Standard Specification for Building Brick (Solid Masonry Units Made from Clay or Shale).

[50] Saboya F., Xavier G., Alexandre J. (2007). The use of the powder marble by-product to enhance the properties of brick ceramic. Constr. Build. Mater..

[51] More A.S., Tarade A., Anant A. (2014). Assessment of suitability of fly ash and rice husk ash burnt clay bricks. Int. J. Sci. Res. Publ..

[52] Ukwatta A., Mohajerani A., Eshtiaghi N., Setunge S. (2016). Variation in physical and mechanical properties of fired-clay bricks incorporating ETP biosolids. J. Clean. Prod..

[53] Vračević M., Ranogajec J., Vučetić S., Netinger I. (2014). Evaluation of brick resistance to freeze thaw cycles according to indirect procedures. Gradevinar.

[54] Davis A.G., Ansari F., Gaynor R.D., Lozen K.M., Rowe T.J., Caratin H., Heidbrink F.D., Malhotra V.M., Simons B., Carino N.J. (1998). Nondestructive Test Methods for Evaluation of Concrete in Structures.

[55] Shetty M. (2005). Concrete Technology.

[56] Malhotra V.M. (2004). Handbook on Nondestructive Testing of Concrete.

[57] Koroth S.R., Fazio P., Feldman D. (1998). Evaluation of Clay Brick Durability Using Ultrasonic Pulse Velocity. J. Arch. Eng..

